# Characteristics and antioxidant activities of seed oil from okra (*Abelmoschus esculentus* L.)

**DOI:** 10.1002/fsn3.3924

**Published:** 2024-01-02

**Authors:** Gangjun Guo, Wenting Xu, Haiqing Zhang, Xiaojing Hu, Yuqin Chen, Xiyong He, Kechang Huang, Shangxuan Ma, Jiarong Fu

**Affiliations:** ^1^ Yunnan Institute of Tropical Crops Jinghong China; ^2^ College of Notoginseng Medicine and Pharmacy Wenshan University Wenshan China; ^3^ Dehong Tropical Agriculture Institute of Yunnan Ruili China

**Keywords:** antioxidant activities, correlation analysis, fatty acids, okra seed oil, tocopherol, total phenol

## Abstract

To investigate the potential functional properties and added value of okra seed oil and provide a scientific basis for further industrial development and production of okra seed oil, its fatty acid profile, total phenolic, fat‐soluble vitamin composition, mineral element composition, and antioxidant activities were examined in this study. Also, correlations between bioactive components and the antioxidant activities of okra seed oil were explored. The study results show that okra seed oil contains 12 types of fatty acids, 65.22% of which are unsaturated acids, and among these unsaturated acids, linoleic acid (43%) and oleic acid (20.16%) are two dominant acid types. Compared with walnut oil and peanut oil, okra seed oil contains relatively high total phenols, fat‐soluble vitamins, and a variety of essential mineral nutrients, with a total phenolic content (TPC) of 959.65 μg/mL, a total tocopherol content of 742.71 μg/mL, a vitamin A content of 0.0017 μg/100 mL, a vitamin D content of 1.44 μg/100 mL, and a vitamin K_1_ content of 52.54 ng/100 mg. Also, okra seed oil exhibits better scavenging activities on hydroxyl (IC_50_ = 0.50 mg/mL) and ammonium salt (ABTS) free radicals (IC_50_ = 6.46 mg/mL) and certain reducing power (IC_50_ = 17.22 mg/mL) at the same concentration. The scavenging activities of okra seed oil on hydroxyl radicals and ABTS radicals, as well as its reducing power, are significantly correlated with its contents of total phenol, total tocopherol, α‐tocopherol, and γ‐tocopherol (*p* < .01). These results show that okra seed oil is rich in bioactive substances, thus presenting great nutritional potential.

## Graphical abstract

Okra seed oil is rich in beneficial components (unsaturated fatty acids, phenolics, and tocopherols) and has stronger antioxidant activities, which lays the foundation for its development as an edible oil.

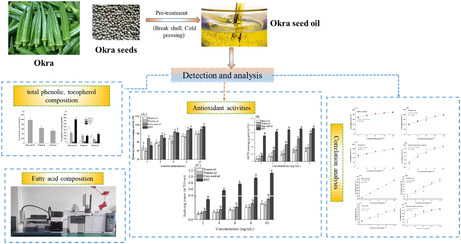

## 1 INTRODUCTION

Originating in Africa, okra (*Abelmoschus esculentus* L.) is an annual multi‐use and high‐value herb type of Malvaceae (also known as okra clip and Crotalaria). Also, okra is a type of vegetable crop that is rich in such nutrients as sugar, protein, minerals, vitamins, and dietary fiber (Naveed et al., 2009). Containing quercetin, sterol, and other components with medicinal value, it is a plant that can be used as medicine and food (Yao et al., 2021). Therefore, it is favored by people as a new type of healthy vegetable (Anwar et al., 2020). Okra is widely distributed and cultivated in Africa, Asia, Southern Europe, and America. Introduced to China in the early 1990s, it is currently distributed and cultivated in South China and North China (Çalışır et al., 2005). The roots, pods, flowers, and seeds of okra can be used in the treatment of spleen deficiency, fatigue, and intestinal dryness with constipation, showing such biological activities as antioxidant, anti‐tumor, anti‐fatigue, anti‐inflammatory, and analgesic activities (Esan et al., 2017; Ma et al., 2021; Xia et al., 2015).

As a new type of healthy vegetable, okra has its nutritional value primarily coming from its seeds (Adelakun et al., 2009). It has been reported that mature okra seeds can be roasted and ground into fine powder to be used as additives or ingredients for Turkish coffee (Adetuyi & Ibrahim, 2014). Round white seeds of immature okra fruits can be primarily used as fresh (or quick‐frozen) food, with abundant dietary fiber, and other bioactive nutrients (Karrar et al., 2021). Some reports have shown that okra seeds are rich in high‐quality proteins and oils that are mainly composed of linoleic acids (Gemede et al., 2015; Xu et al., 2020). Greek okra seeds are a potential source of edible vegetable oils. With an oil content of 15.9%–20.7%, these seeds are rich in unsaturated fatty acids essential for human nutrition (Andras et al., 2005). Studies have also shown that okra seeds have an oil content of around 20%–40% (Benchasr, 2012). Oil yields of okra seeds are closely related to the oil extraction methods that are applied (Dong et al., 2014). Meanwhile, okra seeds contain a wide variety of phenolic compounds, the main components of which are oligocatechins (2.5 mg/g) and flavonol derivatives (3.4 mg/g) (Arapitsas, 2008; Huang et al., 2007). In vitro experiments show that the antioxidant performance of edible oils can be used as an indirect index to evaluate their quality. Tocopherols, polyphenols, and pigments are important components of natural vegetable oils (Anwar et al., 2020). Studies have shown that okra seed powder presents a good DPPH free radical scavenging ability (Adelakun et al., 2009). Also, the antioxidant activities of okra seed oil have gradually become a hot topic of research because of their important physiological effects in resisting cancer, cardiovascular diseases, and aging.

In recent years, with the growth of the population, the development of a healthy oil industry, and the increasing demand for vegetable oils, much attention has been paid to various extracted oils from plant seeds, with some underutilized and new resources of vegetable oils deeply explored (Gemede et al., 2015). Okra seeds are a good source of edible refined oils, with suitable preparation processes in China. The development and production of okra seed oil are expected to alleviate the present shortage of edible oils in the world (Anwar et al., 2010). In spite of their small contents in edible vegetable oils, okra seeds are closely related to the quality of edible vegetable oils and directly affect their functionality and oxidation stability. However, less research has been conducted on okra seed oil, which has emerged in the Chinese market in recent years. Although a lot of research has been performed on the nutritional value of okra, especially the nutritional value of gum polymers in okra seeds, the anti‐oxidation capacity of okra seed oil has not yet been deeply investigated. Also, there is limited content determination and antioxidant activity of fatty acids, total polyphenols, and tocopherols in extracted okra seed oil suitable for industrialized production. In this study, through gas chromatography (GC) and gas chromatography–mass spectrometry (GC–MS), the fatty acid composition of okra seed oil was analyzed, with its contents of total polyphenols and tocopherols identified. Also, its scavenging abilities on hydroxyl free radicals and ABTS free radicals, as well as its reducing power, were investigated. Finally, relationships between the contents of bioactive substances and antioxidant activities were evaluated. In summary, this study aims to provide a theoretical and technical basis for the industrialized development and production of okra seed oil.

## 2 MATERIALS AND METHODS

### 2.1 Standard reagents

The standard α‐, γ‐ and δ‐tocopherol reagents used in this study were supplied by Tianjin Kemiou Chemical Reagent Co., Ltd. (Tianjin, China). Standard reagents of 2,2‐azinobis(3‐ethylbenzothiazoline‐6‐sulfonic acid), ammonium salt (ABTS) and 2,6‐di‐tert‐butyl‐4‐methylphenol (BHT) were supplied by Beijing Putian Tongchuang Biotechnology Co., Ltd. (Beijing, China). Anhydrous ethanol, potassium hydroxide, and BHT were purchased from Sinopharm, which were AR of grade; methyl tert butyl ether, n‐hexane, and methanol were purchased from CNW, which were of HPLC grade. Also, regents of gallic acid monohydrate were supplied by Sinopharm Chemical Reagent Co., Ltd. (Shanghai, China). Other analytical‐grade chemical reagents (purity >90%), such as folin–Ciocalteu reagent, ferrous sulfate heptahydactivity reagent, salicylic acid reagent, and sodium dihydrogen phosphate reagent, were supplied by Tianjin Fuchen Chemical Reagent Co., Ltd. (Tianjin, China).

### 2.2 Preparation of okra seed oil, walnut oil, and peanut oil

Okra seeds used in this study were collected from an experimental base of the Yunnan Institute of Tropical Crops (Jinghong city, Xishuangbanna prefecture, Yunnan province, China), and walnuts and peanuts were purchased from the local market. Ten kilograms of okra seeds were dried in the sun until their water contents dropped to less than 4%. Kernels of okra seeds, walnuts, and peanuts were crushed with a cereal grain milling machine (Guangzhou, China) and then pressed with a cold‐pressing machine (KOMET, D85‐1G, Germany) under the following specific parameters: oil press temperature – 62°C; turn head – D15; turn speed – 20 r/min; and pressure – 40 MPa.

### 2.3 Analysis of fatty acid composition

#### 2.3.1 Extraction and methyl esterification of fatty acids

Okra seed oil with a volume of 50 mL was mixed with 20 mL of sulfuric acid methanol solution with a concentration of 1% and refluxed in a water bath until its oil beads disappeared (for about 5 h). Then, after the reaction product cooled down to room temperature, distilled water with a volume of 60 mL was added to it, along with equal volumes of diethyl ether extracted from the reaction product three times. After that, the extracted diethyl ether was washed with neutral water and dried with anhydrous sodium sulfate, with some parts of the ether evaporated to obtain a fatty acid methyl ester ether solution for further use. Then, the reaction product was stored in a dark glass vial at 4°C for further analysis.

#### 2.3.2 GC/GC–MS analysis

Gas chromatography and GC–MS experimental devices (GC: 6890N Agilent Technologies, USA; GC–MS: HP6890GC/5973MS Agilent Technologies, USA) were used to identify the fatty acid composition of okra seed oil. In the GC experiment, an HP‐5 capillary column with a size of 30 m × 0.32 mm × 0.25 μm was used, with a carrier gas of helium at a flow rate of 1.5 mL/min. The oven temperature was initially held at 150°C, then increased to 280°C with a heating increase rate of 3°C/min, and then kept at 280°C for 5 min. Then, an oil sample with a volume of 1 μL was injected into the oven at 250°C with a split ratio of 50:1. In the GC–MS experiment, an HP‐5MS capillary column with a size of 30 m × 0.25 mm × 0.25 μm was used, with a carrier gas of helium at a flow rate of 1.0 mL/min. The oven temperature was initially held at 150°C, then increased to 260°C with a heating increase rate of 3°C/min, and then kept at 260°C for 5 min. Then, an oil sample with a volume of 0.5 μL was injected into the oven at 250°C with a split ratio of 30:1. In both experiments, mass spectra of electronic ionization and chemical ionization were recorded. Data within the range of 35–500 m/z were obtained through the HRMS system with an electronic ionization energy of 70 eV. A Wiley7n.l mass spectral library search method was used to identify unknown compounds. The relative quantification of fatty acids was carried out by gas chromatography area normalization.

### 2.4 Identification of total phenolic content

Folin–Ciocalteu reagents were used to identify the TPCs of oils with the slightly modified literature method (Gemede et al., 2018; Seyrekoglu et al., 2022). Okra seed oil, walnut oil, and peanut oil with a volume of 5 mL were separately measured, and then ethanol with a volume of 10 mL was added to each oil to perform ultrasonic extraction for 1 h. A transparent solution with a volume of 100 mL was filled into a 100 mL volumetric flask for later testing. A test solution with a volume of 5 mL was obtained and added with 1 mL of Folin–Ciocalteu reagent and 3 mL of 7.5% sodium carbonate solution for color development. Two hours later, a 722 UV–VIS spectrophotometer (Jinghua Technology Instrument Co., LTD, Shanghai) was used to measure the absorbance of oil samples at a wavelength of 765 nm for TPC evaluation. Also, gallic acids were used as standard reagents (*Y* = 0.014*X* + 0.0157, *R* ^2^ = .9990), with results of gallic acid equivalents (GAE) per gram of oil extract calculated.

### 2.5 Analysis of fat‐soluble vitamins

Methanol was added to different oil samples, with their volumes adjusted to 100 mL. With 0.22 μm organic filtration membranes, their supernatants were obtained and placed in a refrigerator at 4°C for further testing. Samples with a volume of 3 μL were injected into a high‐performance liquid chromatography device (HPLC; Agilent Zorbax SB) with a column of C18 (250 × 4.6 mm, 5 μm). A mobile phase of acetonitrile was used with distilled water (98:2 v/v) at a flow rate of 1.0 mL/min. The fluorescence detector was set at a wavelength parameter of 325, 264, and 292 nm for the vitamin A, vitamin D, and vitamin E analysis, respectively. Quantification was performed through external standardization with a standard curve from standard stock solutions (Sigma‐Aldrich).

Vitamin K: 500 μL of water was added to the sample (0.5 g); after full mixing, 2 mL of n‐hexane was added, shaken well, extracted for 30 min, and then centrifuged at 12,000 rpm for 10 min to obtain the supernatant. Then it was passed through the alumina column, and 2 mL of n‐hexane was added to leach the supernatant. The extract was collected with 2 mL of eluent (n‐hexane:ethyl acetate = 9:1), and dried with nitrogen, then redissolved with 200 μL of methanol for further analysis. The sample extracts were analyzed using an LC–MS/MS system (UPLC, Vanquish; MS, QE) with Waters ACQUITY UPLC HSS T3 (2.1 × 50 mm, 1.8 μm). LC Conditions: the column temperature, flow rate, and injection volume were 40°C, 0.4 mL/min, and 2 μL, respectively. Solvent system–water (0.1% acetic acid): acetonitrile (0.1% acetic acid). Gradient program–80:20 V/V at 0 min, 80:20 V/V at 1.0 min, 0:100 V/V at 4.0 min, 0:100 V/V at 7.0 min, 80:20 V/V at 7.1 min, and 80:20 V/V at 11.0 min. LC–MS/MS conditions: HRMS data were recorded on a Q Exactive hybrid Q–Orbitrap mass spectrometer equipped with a heated ESI source (Thermo Fisher Scientific) utilizing the SIM MS acquisition methods. The ESI source parameters were set as follows: spray voltage, 3 kV; sheath gas pressure, 40 arb; aux gas pressure, 10 arb; sweep gas pressure, 0 arb; capillary temperature, 320°C; and aux gas heater temperature, 350°C. Data were acquired on the Q‐Exactive using Xcalibur 4.1 (Thermo Scientific) and processed using TraceFinder™ 4.1 Clinical (Thermo Scientific).

### 2.6 Mineral elements

Five milliliter of nitric acid and 1 mL of perchloric acid were mixed into a Teflon tank and placed on an electric heating plate for heating (130–150°C). Increased the electric temperature to 180°C to continue digestion after a large amount of brown smoke disappeared. If the digestion solution turned brown and black, add a small amount of nitric acid appropriately until white smoke emerges. After cooling (the digestion solution was colorless and transparent or slightly yellow), the digestion solution was transferred to a 50 mL volumetric bottle, and the beaker was washed several times with a small amount of water. The lotion was combined in a volumetric bottle, set to scale, and then mixed and filtered. The filtrate was determined by an inductively coupled plasma mass spectrometer (Thermo Fisher, iCAP RQ). The external standard method was used for quantification.

### 2.7 Antioxidant activity

#### 2.7.1 Evaluation of radical scavenging ability with a hydroxyl assay

The hydroxyl radical scavenging activities of oil samples were evaluated based on a slightly modified method previously described (Tai et al., 2014; Zhao et al., 2014). Solutions of oil samples mixed with BHT ethanol (1 mL) under different concentrations (2–10 mg/mL) were added into 10 mL test tubes separately, with the successive addition of distilled water (5 mL), FeSO_4_ solution (0.1 mL, 6 mmol/L), and salicylic acid solution (0.2 mL, 6 mmol/L). Then, an H_2_O_2_ solution (0.1 mL, 1 mmol/L) was added into each tube to start the reaction, and after that, each test tube was incubated at 37°C in a water bath for 30 min. An absorbance wavelength of 515 nm was measured in each solution. With butylated hydroxytoluene (BHT) used as a positive control, the hydroxyl radical scavenging capacities of oils can be calculated using the following formula:

$$\text{Hydroxyl radical scavenging activity}\;\left(\%\right)=\left[\frac{\left(A_{i}-A_{0}\right)}{A_{i}}\right]\times 100$$

where *A*_*i*_ represents the absorbance of the test sample (oil mixed with BHT) and *A*_0_ represents the absorbance of the control (with no oil).

Inhibitory concentrations (IC_50_) of oil samples under which hydroxyl radicals are scavenged by 50% were assessed through linear regression of their concentration‐response curves. With synthetic commercial antioxidants (BHT) used as a standard, tests were performed three times.

#### 2.7.2 Evaluation of free radical scavenging ability through the ABTS assay

The ABTS free radical scavenging abilities of oil samples were evaluated based on a slightly modified method previously described (Ehiobu et al., 2021). Solutions of potassium persulfate (440 μL, 140 mmol/L) and ABTS (25 mL, 7 mmol/L) were mixed and reacted in a dark glass vial for 12–16 h. Before the assessment, an ABTS solution was diluted with anhydrous ethanol until its absorbance value reached 0.700 ± 0.002 (at a wavelength of 734 nm). Then, ABTS‐diluted solutions with a volume of 4 mL were mixed with oil samples (3 mL) at different concentrations (2–10 mg/mL) separately. After that, an absorbance wavelength of 734 nm, marked as *A*_*j*_, was measured in each solution. Then, with the application of anhydrous ethanol instead of oil samples, the above process was repeated, with the measured absorbance wavelength recorded as *A*_0._ The scavenging activities of oil samples on ABTS radicals can be calculated using the following equation.

$$\text{Scavenging activity}\;\left(\%\right)=\left[\frac{\left(A_{0}-A_{i}\right)}{A_{0}}\right]\times 100$$

Inhibitory concentrations (IC_50_) of oil samples at which ABTS radicals are scavenged by 50% were assessed through linear regression of their concentration–response curves. With the synthetic commercial antioxidant BHT used as a standard, tests were conducted three times.

#### 2.7.3 Evaluation of reduced power

Based on the Prussian blue method described in reference literature with slight modifications (Kossah et al., 2011; Saad et al., 2021), the reducing power of oil samples was assessed in this study. Solutions of oil samples mixed with BHT ethanol under different concentrations (2–10 mg/mL, 1 mL) were added with a phosphoric acid buffer (2.5 mL; pH = 6.6) and a potassium ferricyanide solution (2.5 mL, 1%) and reacted in a water bath for 20 min at 50°C. Then, each solution was added with trichloroacetic acid (2.5 mL, 10%) under a cool condition, shaken well, and centrifuged at 3000 r/min for 10 min. After that, a supernatant volume of 2.5 mL was taken in each solution, mixed with deionized water (2.5 mL) and 0.1% FeC1_3_ (0.5 mL) in a 10 mL test tube, and reacted for 10 min. A UV–visible spectrophotometer with a wavelength of 700 nm was used to measure the absorbance values of these solutions, with the reducing power of oil samples expressed with their corresponding absorbance values measured. With BHT used as a positive control, tests were performed three times. Through linear regression analysis, IC_50_ values (defined as the effective concentration of solution under which an absorbance value of 0.5 at the wavelength of 700 nm is reached) of oil samples were obtained.

### 2.8 Statistical analysis

In this study, SPSS 19.0 (SPSS Inc., Chicago, IL) was used to perform the Duncan's test (*p* < .05), with Origin 9.0 (Origin Lab Co., Northampton, MA, USA) used in diagram plotting. All experiments were conducted independently three times, with results expressed as the mean ± standard error. A two‐tailed Pearson's correlation test was conducted to identify the correlations among the mean values obtained. Also, an REG process method was applied to perform the multiple linear regression analyses in this study. Finally, SAS version 9.2 for Windows (SAS Inc., Cary, NC, USA) was used for all statistical analyses conducted in this study.

## 3 RESULTS AND DISCUSSION

### 3.1 Fatty acid composition

**FIGURE 1:**
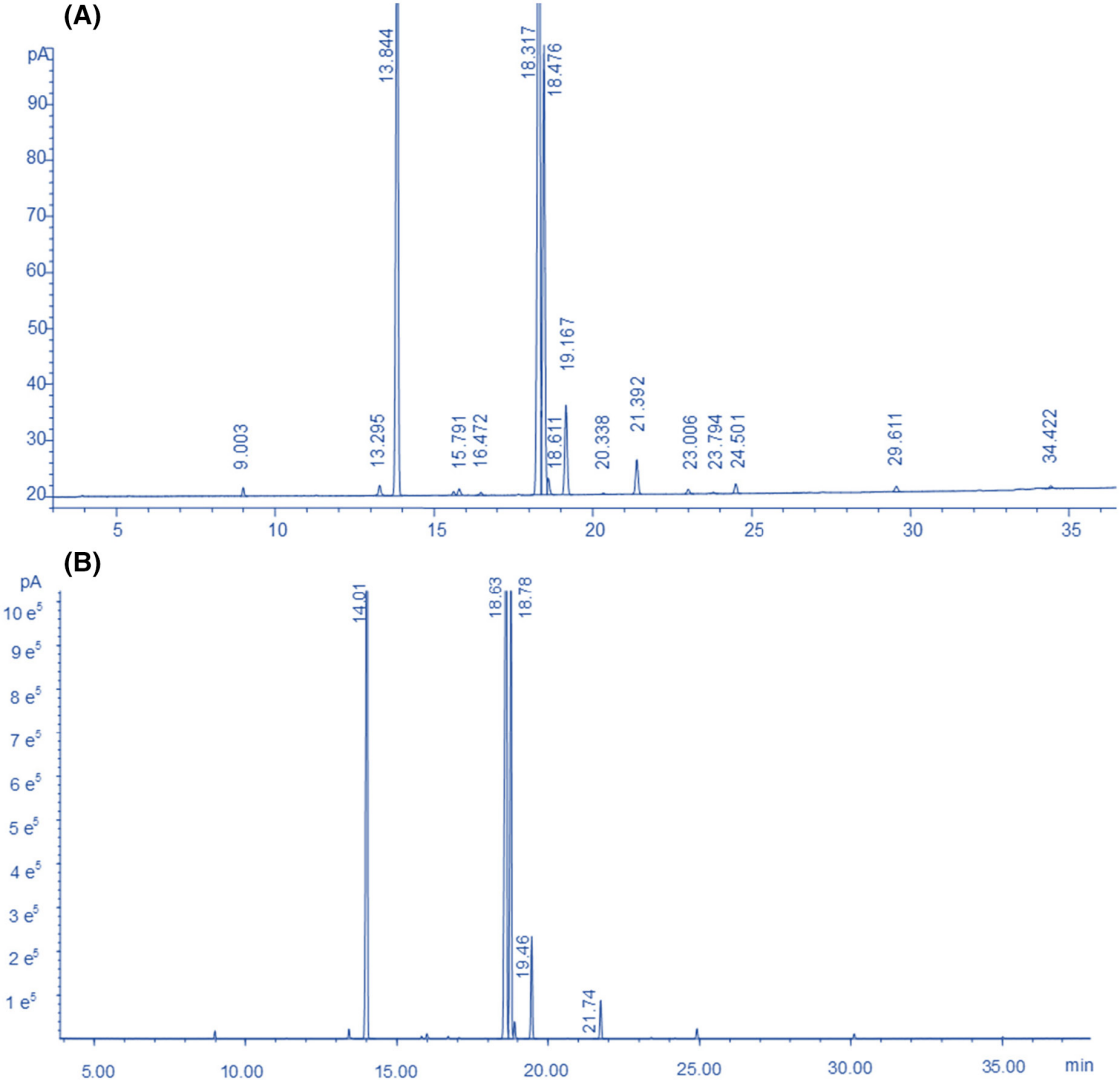
GC chromatogram (A) and GC–MS total ion current chromatogram (B) of okra seed oil.

“Lipids and oils are important storage forms of carbon in many seeds” (Zhang et al., 2014). The fatty acid composition (expressed as a percentage of total fatty acids, g/100 g) of okra seed oil obtained through GC/GC–MS analyses is presented in Figure 1 and Tables 1 and 2. Okra seed oil has an approximate 34.28% concentration of saturated fatty acids (SFA) (mainly including tetradecanoic acid, palmitic acid, heptadecanoic acid, octadecanoic acid, eicosanoic acid, docosanoic acid, and tetracosanoic acid), and a 65.53% concentration of unsaturated fatty acids (UFA) (mainly including linoleic acid, oleic acid, and 10‐nonadecenoic acid, with concentrations of 43.00%, 20.16%, and 1.60%, respectively). The above‐mentioned concentration of SFA is close to those concentrations of seed oils (*Hibiscus esculentus*) reported by Los Baños (35.30%) (Murata et al., 2003), Pakistan (36.59%–37.66%) (Anwar et al., 2010, 2011) and Greece (37.60%) (Andras et al., 2005). Anwar et al. (2010, 2011) reported that the contents of linoleic acids in seed oils (*H. esculentus*) from Pakistan fall into a range of 29.90%–31.70%, which is close to the percentage proposed in this article (34.00%).

**TABLE 1:** Fatty acids composition of okra seed oil, walnut oil, and peanut oil.

Oil sample	Extraction method	Origin	Fatty acid/%																				2‐Octyl‐cyclopropaneoctanoic acid	2‐Hexyl‐cyclopropaneoctanoic acid	References
			C_12:0_	C_14:0_	C_15:0_	C_16:0_	C_16:1_	C_17:0_	C_17:1_	C_18:0_	C_18:1_	C_18:2_	C_18:3_	C_19:1_	C_20:0_	C_20:1_	C_20:3_	C_20:5_	C_21:0_	C_22:0_	C_22:6_	C_24:0_			
Okra seed	Cold‐pressing	China	ND	0.27	ND	28.75	0.46	0.13	0.31	3.96	20.16	43.00	ND	1.6	0.48	ND	ND	ND	ND	0.27	ND	0.11	ND	ND	Present study
	N‐hexane	Pakistan	ND	0.21	ND	30.23	0.28	0.15	ND	4.93	29.09	30.31	ND	ND	0.73	ND	ND	ND	ND	0.34	ND	ND	1.92^a^	0.25^a^	Anwar et al. (2010)
Walnut	Hexane/isopropanol (3:2, v/v)	Ireland	ND	0.13	ND	6.70	0.23	ND	ND	2.27	21.00	57.46	11.58	ND	0.08	ND	ND	0.06	ND	0.07	ND	ND	ND	ND	Maguire et al. (2004)
	Hexane	Turkey	ND	ND	0.52	6.70	0.05	ND	ND	2.58	17.22	59.83	12.65	ND	0.09	0.18	ND	ND	ND	ND	0.18	ND	ND	ND	Gecgel et al. (2011)
	Cold‐pressing	Poland	ND	0.04	ND	5.11	0.07	0.04	ND	2.57	16.93	58.54	14.38	ND	0.17	0.97	ND	ND	ND	ND	ND	ND	ND	ND	Turek et al. (2021)
Peanut	Screw‐pressing	Turkey	ND	ND	ND	9.37	ND	ND	ND	3.73	55.33	23.69	ND	ND	1.83	1.57	ND	ND	ND	3.25	ND	1.62	ND	ND	Konuskan et al. (2019)
	Hexane/isopropanol (3:2, v/v)	Ireland	ND	0.03	ND	11.08	0.15	ND	ND	2.66	38.41	44.60	0.58	ND	1.57	ND	0.02	0.02	ND	0.10	0.75	ND	ND	ND	Maguire et al. (2004)
	Cold‐pressing	India	0.03	0.15	ND	14.87	0.13	0.14	ND	6.26	49.60	20.38	0.31	ND	3.44	1.80	ND	ND	1.89	ND	ND	ND	ND	ND	Ananth et al. (2019)

Abbreviation: ND, Not detected.

^a^ The relative percentage of 2‐octyl‐cyclopropaneoctanoic acid is 1.92%, and 2‐hexyl‐cyclopropaneoctanoic acid 0.25% in the literature of Anwar et al. (2010).

**TABLE 2:** Summary of the important fatty acid parameters of okra seed oil, walnut oil, and peanut oil.

Oil sample	Extraction method	Origin	Polyunsaturated fatty acids/%	Monounsaturated fatty acids/%	Total saturated fatty acids/%	Total unsaturated fatty acids/%	Ratio unsaturated/saturated	References
Okra seed	Cold‐pressing	China	43.00	22.53	33.97	65.53	1.93	Present study
	N‐hexane	Pakistan	30.31	31.54	36.59	61.85	1.69	Anwar et al. (2010)
Walnut	Hexane/isopropanol (3:2, v/v)	Ireland	69.10	21.23	9.25	90.33	9.77	Maguire et al. (2004)
	Hexane	Turkey	72.66	17.45	9.89	90.11	9.11	Gecgel et al. (2011)
	Cold‐pressing	Poland	72.92	17.97	7.93	90.89	11.46	Turek et al. (2021)
Peanut	Screw‐pressing	Turkey	23.69	56.90	19.80	80.59	4.07	Konuskan et al. (2019)
	Hexane/isopropanol (3:2, v/v)	Ireland	45.97	38.56	15.44	84.53	5.47	Maguire et al. (2004)
	Cold‐pressing	India	20.69	51.53	26.78	72.22	2.70	Ananth et al. (2019)

### 3.2 Fat‐soluble vitamin composition

**FIGURE 2:**
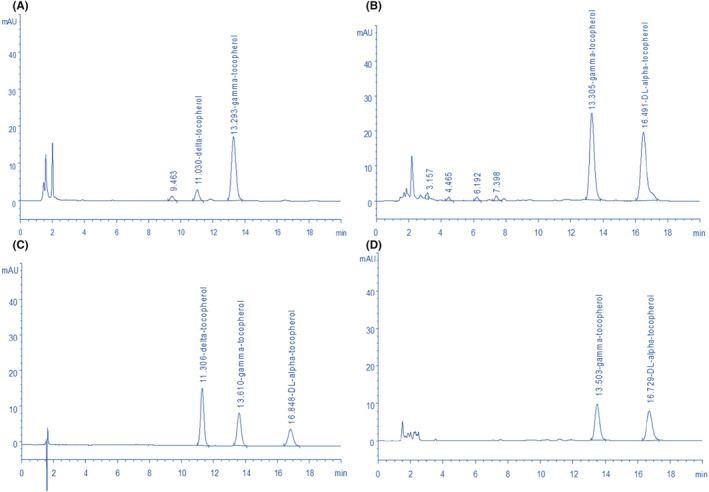
The HPLC chromatogram of α‐, γ‐, and δ‐tocopherol. (A) the standards of α‐, γ‐, and δ‐tocopherol; (B) okra seed oil; (C) walnut oil; (D) peanut oil.

**FIGURE 3:**
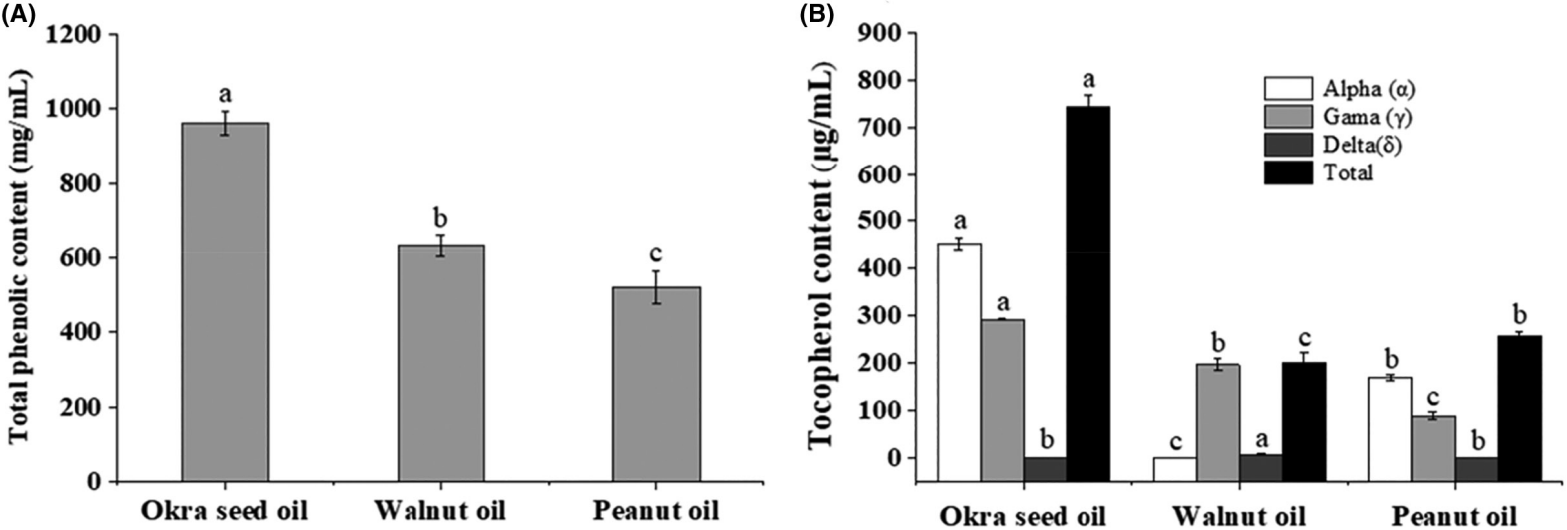
Total phenolic contents (A), contents of α‐, γ‐, δ‐tocopherol, and total tocopherol (B) in okra seed oil, walnut oil, and peanut oil. Values are means of triplicates ± standard deviation (SD). The bars with different letters among samples are significantly different (*p* < .05) by Duncan test.

As a good natural fat‐soluble vitamin, tocopherol can protect vulnerable substances in the human body from oxidation and reduce the occurrence of lipid peroxidation. Several studies have revealed that “tocopherol can regulate lipid oxidation, scavenge free radicals and protect cells from oxidant damage” (Rodríguez et al., 2021). As shown in Figures 2 and 3B and Table 3, our study indicates that the vitamin K_1_, α‐tocopherol, and γ‐tocopherol contents in okra seed oil are 52.54 ng/100 mg, 450.79 μg/mL, and 291.92 μg/mL, respectively, which are higher than those contents in walnut oil (10.43 ng/100 mg, 0.00 μg/mL, and 195.72 μg/mL) and peanut oil (5.52 ng/100 mg, 168.01 μg/mL and 87.48 μg/mL), and walnut oil is rich in vitamin D (2.31 μg/100 mL), which is significantly higher than okra oil and peanut oil, but does not contain vitamin A. Peanut oil is rich in vitamin A, and its content was 0.058 μg/100 mL, significantly higher than okra oil (0.0017 μg/100 mL). Vitamin K_2_ is absent in all three oils. Also, the total tocopherol content in okra seed oil is 742.71 μg/mL, which is much higher than those contents in walnut oil (200.90 μg/mL) and peanut oil (255.49 μg/mL). δ‐tocopherol is absent in okra seed oil and peanut oil, with a content of 5.18 μg/mL in walnut oil. Anwar et al. (2011) reported that seed oils of two Okra varieties of Sabz Pari and Punjab‐8 (*H. esculentus*) cultivated under similar environments present tocopherol (α, γ, and δ) contents ranging from 653.0 to 696.5 mg/kg, 2.13 to 3.33 mg/kg, and 1.01 to 1.11 mg/kg, respectively. The α‐tocopherol content of okra seed oil obtained in this research is considerably lower than that of the seed oils mentioned above (*H. esculentus*) (653–696 mg/kg). However, the α‐tocopherol content in okra seed oil obtained in this study is higher than that content (320.1 mg/kg) in cold‐pressed okra oil reported by Topkafa (2016). These differences in α‐tocopherol contents could result from the differences in okra species, their growing environments, and extraction methods. This study shows that okra seed oil is rich in α‐tocopherols and γ‐tocopherols, all of which may play an important role in its antioxidant activities.

**TABLE 3:** The content of fat‐soluble vitamins (VA, VD, VK) in okra seed oil, walnut oil, and peanut oil.

Fat‐soluble vitamins	Okra seed oil	Walnut oil	Peanut oil
VA (μg/100 mL)	0.002 ± 0.00^b^	–	0.058 ± 0.00^a^
VD (μg/100 mL)	1.44 ± 0.13^b^	2.31 ± 0.06^a^	1.39 ± 0.036^b^
VK1 (ng/100 mg)	52.54 ± 0.48^a^	10.43 ± 0.13^b^	5.52 ± 0.024^c^

*Note*: Superscript letters a, b, and c indicate a significant difference in the same line (*p* < .05). That is, an analysis of the significance of differences between different vegetable oils.

### 3.3 Total phenolic content

Phenols, which are widely found in the leaves, seeds, roots, stems, and rinds of plants, are secondary metabolites generated during plant growth and metabolism. As one active ingredient of plants reflecting their antioxidant activities, phenol plays a very important role in its oxidation resistance (Siddhuraju & Becker, 2003). From Figure 3A, it can be seen that the TPCs of okra seed oil, walnut oil, and peanut oil are 959.65, 631.75, and 522.45 mg/mL, respectively (*p* < .05). Some studies have reported that okra seeds are a good source of total phenols, which are primarily composed of quercetin derivatives, catechins, kaempferol, and flavonol derivatives (Arapitsas, 2008; Huang et al., 2007). This research shows that the TPC of okra seed oil is higher than that of walnut oil and peanut oil, making okra seeds a good source of edible oils.

### 3.4 Mineral elements

As can be seen from Table 4, the three vegetable oils contain various mineral elements such as sodium, phosphorus, potassium, calcium, iron, zinc, and selenium. The content of phosphorus, calcium, and potassium in okra seed oil is 0.063, 0.017, and 0.016 g/kg, which are significantly higher than walnut oil and peanut oil, and the trace elements such as sodium, iron, and selenium are also significantly higher than walnut oil and peanut oil. It can be seen that okra is rich in various nutrients necessary for the human body, and regular consumption is good for health.

**TABLE 4:** Mineral element composition in okra seed oil, walnut oil, and peanut oil.

Element	Okra seed oil (g/kg)	Walnut oil (g/kg)	Peanut oil (g/kg)
Sodium	0.0023 ± 0.0006^a^	0.0030 ± 0.0000^a^	0.0030 ± 0.0000^a^
Phosphorus	0.063 ± 0.0012^a^	0.011 ± 0.0006^c^	0.019 ± 0.0010^b^
Potassium	0.016 ± 0.0015^a^	0.0037 ± 0.0006^b^	0.0017 ± 0.0006^c^
Calcium	0.017 ± 0.0006^a^	0.013 ± 0.0006^b^	0.013 ± 0.0006^b^
Iron	0.0033 ± 0.0006^a^	0.0010 ± 0.0000^b^	0.0010 ± 0.0000^b^
Zinc	0.0013 ± 0.0006^b^	0.0033 ± 0.0006^a^	0.0023 ± 0.0006^ab^
Selenium	0.0063 ± 0.0006^a^	0.0047 ± 0.0006^b^	0.0000 ± 0.0000^c^

*Note*: Superscript letters a, b, and c indicate a significant difference in the same line (*p* < .05). That is, an analysis of the significance of differences between different vegetable oils.

### 3.5 Antioxidant activity

#### 3.5.1 Hydroxyl radical‐scavenging activity

**FIGURE 4:**
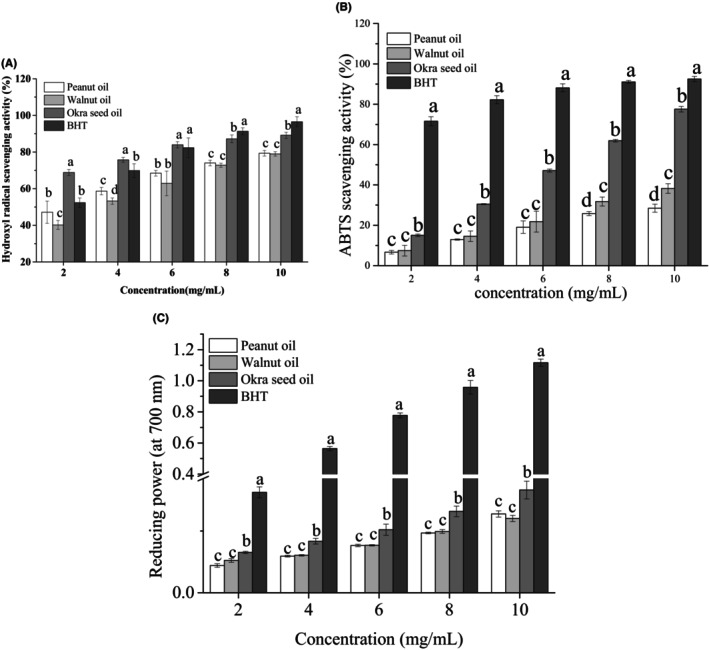
Hydroxyl radical scavenging activity (A), ABTS radical scavenging activity (B), and reducing power (C) of okra seed oil, walnut oil, peanut oil, and BHT. Values are means of triplicates ± standard deviation (SD). The bars with different letters among samples are significantly different (*p* < .05) by Duncan test.

**FIGURE 5:**
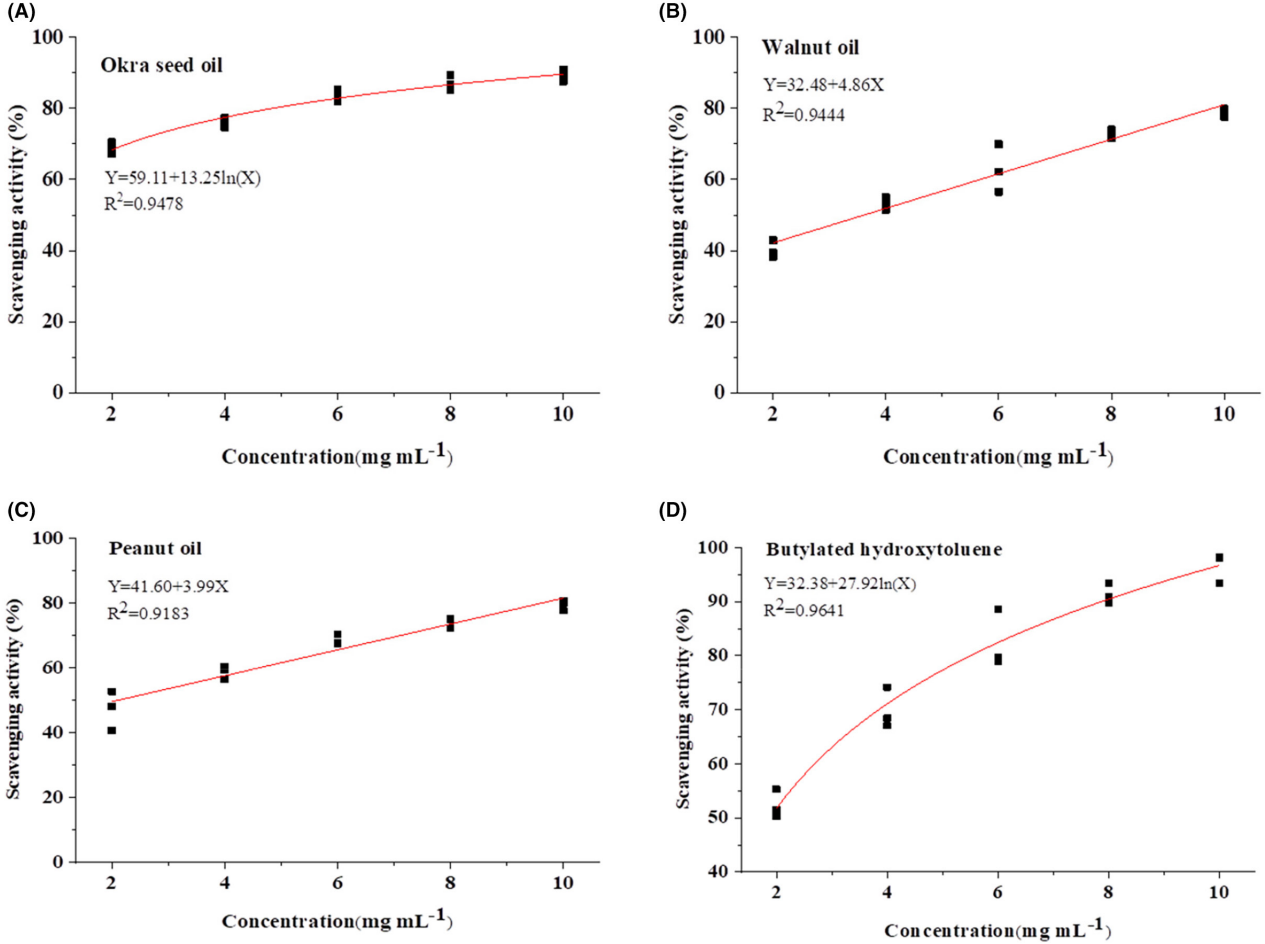
Correlation analysis between hydroxyl radical scavenging activity and concentration of okra seed oil (A), walnut oil (B), peanut oil (C), and BHT (D).

“Okra seeds play a primary role in the free radical scavenging activities of okra” (Xia et al., 2015). However, few studies have been conducted on the anti‐oxidation capacity of okra seed oil. Hydroxyl radical scavenging activity is an important indicator reflecting the antioxidant capacities of substances and is of great significance for antioxidant research (Lu et al., 2021). Figure 4A shows the hydroxyl radical scavenging activities of the oils obtained in this study. Within a concentration range of 2–10 mg/mL in this test, the hydroxyl radical scavenging activities of okra seed oil, walnut oil, peanut oil, and BHT all increased gradually, with correlation coefficients (*R* ^2^) of .9478, .9444, .9183, and .9641, respectively (Figure 5). Okra seed oil presents a significantly higher hydroxyl radical scavenging capacity than those of walnut oil and peanut oil within a concentration range of 2.0–10.0 mg/mL (*p* < .05), which could result from its higher contents of polyphenols, reducing agents, and hydrogen donors than those of walnut oil and peanut oil. Okra seed oil, BHT, peanut oil, and walnut oil present IC_50_ of 0.50, 1.88, 2.11, and 3.60 mg/mL, respectively (Table 5). These results show that okra seed oil exhibits a higher hydroxyl radical scavenging activity than that of walnut oil and peanut oil.

**TABLE 5:** IC_50_ values for antioxidant activity of okra seed oil, walnut oil, peanut oil, and BHT.

Samples (mg/mL)	Hydroxyl radical assay	ABTS radical assay	Reducing power assay
Okra seed oil	0.50 ± 0.08^c^	6.46 ± 0.04^c^	17.22 ± 2.21^c^
Walnut oil	3.60 ± 0.39^a^	12.92 ± 1.58^b^	25.20 ± 0.86^a^
Peanut oil	2.11 ± 0.58^b^	17.10 ± 0.91^a^	22.48 ± 1.10^b^
Butylated hydroxytoluene	1.88 ± 0.22^b^	0.37 ± 0.07^d^	3.49 ± 0.18^d^

*Note*: Superscript letters a, b and c indicate a significant difference in the same line (*p* < .05). That is, an analysis of the significance of differences between different vegetable oils.

#### 3.5.2 ABTS radical scavenging activity

**FIGURE 6:**
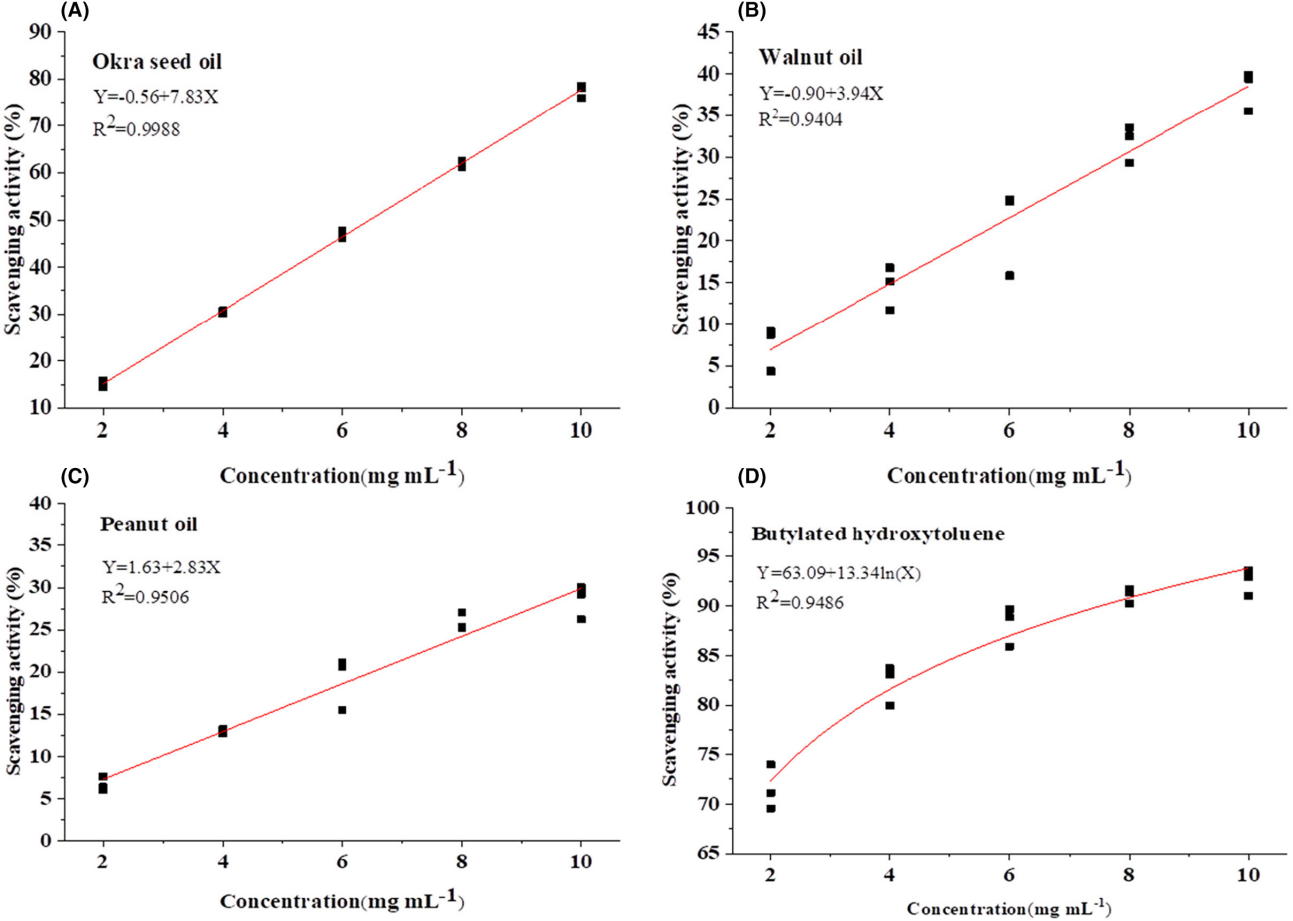
Correlation analysis between ABTS radical scavenging rate and concentration of okra seed oil (A), walnut oil (B), peanut oil (C), and BHT (D).

Figure 4B shows the ABTS radical scavenging capacities of oil samples. With increases in concentrations of okra seed oil, walnut oil, peanut oil, and BHT samples, their percentage inhibition of ABTS radicals also increases, presenting a good dose‐dependent relationship, with correlation coefficients (*R* ^2^) of .9988, .9404, .9506, and .9486, respectively (Figure 6). The ABTS radical scavenging capacity of okra seed oil is significantly higher than that of walnut oil and peanut oil (*p* < .05) and lower than that of BHT (*p* < .05) within the concentration range of 2.0–10.0 mg/mL. IC_50_ values of all oil samples were calculated based on the plotted graphs of percentage scavenging activities against concentrations of oil samples (Table 5). It can be seen that oils with a lower IC_50_ value present a higher radical scavenging potential. Also, okra seed oil presents an IC_50_ range of 6.46 ± 0.04 mg/mL, and peanut oil presents an IC_50_ range of 17.10 ± 0.91 mg/mL (Table 3). Oil samples presented significantly different radical scavenging activities (*p* < .05) in this study, with BHT exhibiting the strongest scavenging activity (lowest IC_50_ value). Also, in this study, okra seed oil presented a relatively strong radical scavenging capacity with an IC_50_ of 6.46 mg/mL, while walnut oil and peanut oil presented relatively weak radical scavenging capacities, with IC_50_ of 12.92 and 17.10 mg/mL, respectively. Similarly, Adetuyi and Ibrahim (2014) and Adelakun et al. (2011) reported that okra seed oil presents a relatively low value of IC_50_ and a relatively strong radical scavenging capacity.

#### 3.5.3 Reducing power

**FIGURE 7:**
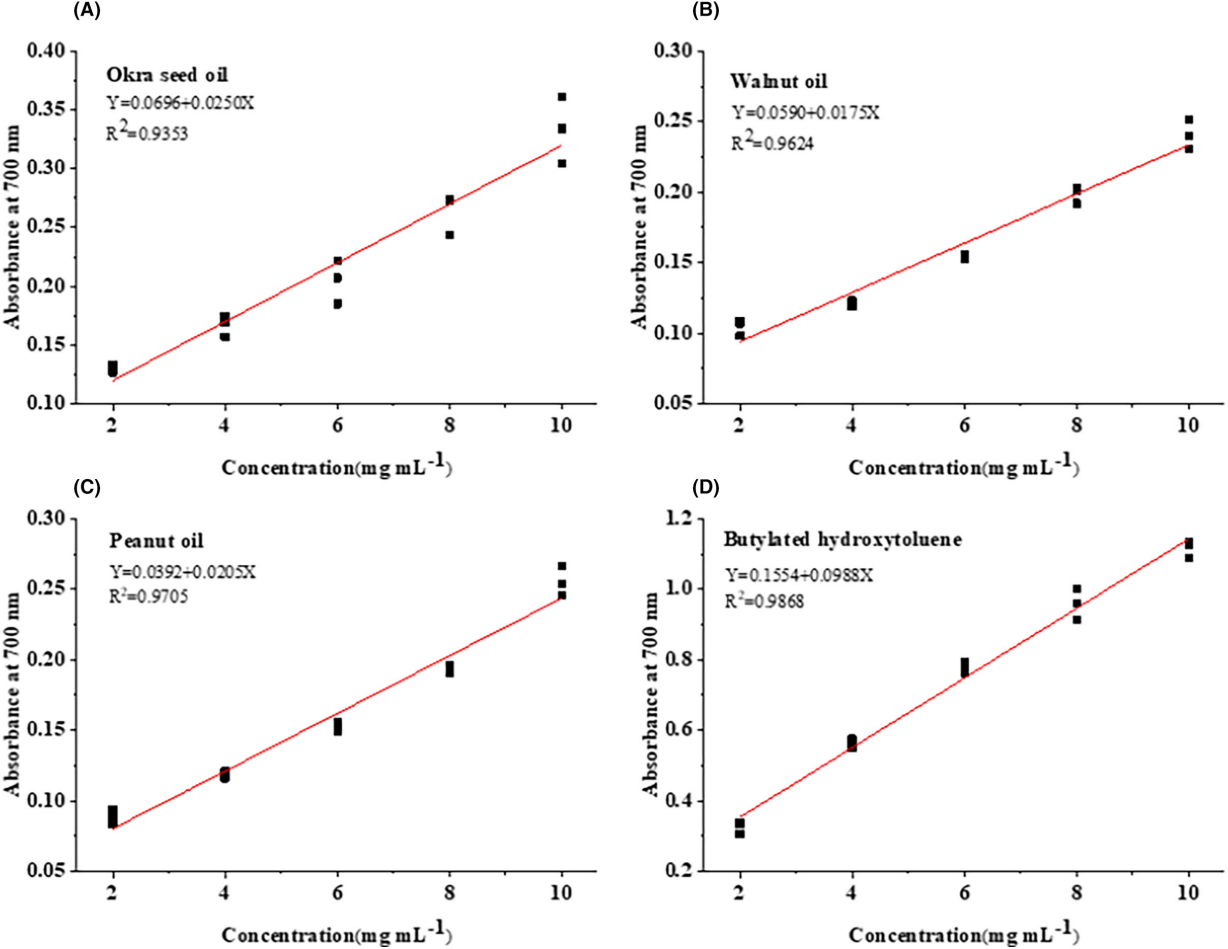
Correlation analysis between reducing powers and concentration of okra seed oil (A), walnut oil (B), peanut oil (C), and BHT (D).

The reducing power of a compound refers to its ability to transfer electrons and serves as a useful indicator of its electron‐donating activity, which is an important mechanism of the phenolic antioxidant reaction (Adetuyi & Ibrahim, 2014). A higher absorbance value of a compound indicates its higher reduction capacity (Do et al., 2014). Figure 4C presents the reducing power of oil samples obtained in this study. It can be seen that, with increases in their concentrations, the reducing power of okra seed oil, walnut oil, peanut oil, and BHT all increases gradually, presenting a good dose‐dependent relationship with *R* ^2^ values of .9353, .9624, .9705, and .9868, respectively (Figure 7). Okra seed oil presents significantly higher reducing power than the reducing power of walnut oil and peanut oil (*p* < .05) and lower reducing power than that of BHT (*p* < .05) within the concentration range of 2.0–10.0 mg/mL. The calculation results of the IC_50_ values of oil samples are listed in Table 5. It can be seen that okra seed oil presents an IC_50_ of 17.22 mg/mL, which is lower than those of walnut oil (25.20 mg/mL) and peanut oil (22.48 mg/mL), and higher than that of BHT (3.49 mg/mL), exhibiting stronger reducing power than that of walnut oil and peanut oil. Similarly, some studies have reported that okra seeds present remarkable reducing power, which can be enhanced after seed fermentation (Adetuyi & Ibrahim, 2014). The reducing power of okra seeds follows a similar changing pattern to those patterns of their TPCs and their hydroxyl and ABTS radical scavenging activities. These results show that the antioxidant capacities of the three different vegetable oil types investigated in this study are closely related to the types and contents of their bioactive components. Furthermore, although okra seed oil has a lower content of UFA than that of walnut oil and peanut oil, it presents stronger antioxidant activity than that of walnut oil and peanut oil. Also, it can be seen that there are no correlations between the contents of UFA, MUFA, and PUFA in oil samples and their antioxidant activities (Zhang et al., 2014). Furthermore, this study shows that the fatty acids in okra seed oil have no effect on its antioxidant activity. Some previous studies have also shown that UFA (i.e. 18:1, 18:2n–6, and 18:3n–3) presents no antioxidant activities in its free forms (Li et al., 2007).

### 3.6 Correlations between the bioactive components of okra seed oil and its antioxidant activity

Experiments conducted in this study show that okra seed oil is rich in fatty acids, phenols, and tocopherols, presenting strong antioxidant activity. However, the specific mechanism of okra seed oil's antioxidant activity remains unclear. With no relevant research report on such mechanisms, some related investigations were carried out in this study. Phenolic molecules are able to donate hydrogen atoms to free radicals or reduce the number of free radicals, making them important antioxidant components for deactivating free radicals. Previous studies have reported that there are linear correlations between the total phenolic and flavonoid contents of oils and their antioxidant capacities (Boulanouar et al., 2013). Table 6 shows the obtained correlations (*R* ^2^) between the antioxidant activity of okra seed oil and its total phenolic, total tocopherol, α‐tocopherol, and γ‐tocopherol contents through those three assays (hydroxyl and ABTS radical scavenging ability and reducing power) conducted in this study. It can be seen that the TPC of okra seed oil presents the strongest correlations with its hydroxyl and ABTS radical scavenging capacities and reducing power, with *R* ^2^ of .8517, .9719, and .9729, respectively. This could be explained by the conjugative effect of the phenolic benzene ring. Under this effect, hydroxyl on the benzene ring serves as an excellent provider of electrons and hydrogen. Also, followed by its contents of γ‐tocopherol (*R* ^2^: .7632, .9595, and .8751) and α‐tocopherol (*R* ^2^: .7302, .8493, and .7362), the total tocopherol content of okra seed oil presents the second strongest correlations with its hydroxyl and ABTS radical scavenging capacities and reducing power, with *R* ^2^ of .8072, .9647, and .8540, respectively. Similarly, previous studies have shown that the total phenolic and total flavonoid contents of different okra organs (including flower, fruit, leaf, and seed) are related to their antioxidant capacity (Gemede et al., 2015). Some previous studies have reported that the phenolic contents of *Achillea arabica* populations are significantly related to their antioxidant activities, indicating that phenolic compounds could play a primary role in their antioxidant capacities (Cirak et al., 2022). Some other studies have shown that total phenolic compounds present strong antioxidant capacities (Bubonja‐Sonje et al., 2011). This study shows that there are weak correlations between the δ‐tocopherol content of okra seed oil and its scavenging capacities of hydroxyl and ABTS radicals, as well as its reducing power(*R* ^2^ = −.070, .034, and .051). A possible reason is that hydroxyl ranks sixth in the chroman ring of tocopherol as a kind of action group, releasing active hydrogen to eliminate free radicals, terminate chain reactions, and exert its antioxidation effect (Korotdova et al., 2004).

**TABLE 6:** Pearson coefficients between antioxidant activities and bioactive components and correlation test of okra seed oil, walnut oil, and peanut oil.

Items	Total phenolic	Total tocopherol	α‐Tocopherol	γ‐Tocopherol	δ‐Tocopherol	Hydroxyl radical	ABTS radical	Reducing power
Total phenolic	1							
Total tocopherol	0.89454**	1						
α‐Tocopherol	0.75057**	0.95569**	1					
γ‐Tocopherol	0.95174**	0.86746**	0.68257**	1				
δ‐Tocopherol	0.10646	−0.28625	−0.55437*	0.22607	1			
Hydroxyl radical	0.85166**	0.80715**	0.73023**	0.76932**	−0.07044	1		
ABTS radical	0.97194**	0.96472**	0.84928**	0.95954**	−0.03368	0.83623**	1	
Reducing power	0.97285**	0.85398**	0.73621**	0.87507**	0.05147	0.85773**	0.92004**	1

* Significant correlation, *p* < .05.

** Extremely significant correlation, *p* < .01.

### 3.7 Multiple regression analyses of the bioactive components and antioxidant activities of oils

Usually, the strong antioxidant activities of vegetable and seed oils are the result of ingredients combined together. Different combinations of bioactive ingredients could lead to different antioxidant activities of these oils (Jiang et al., 2015). In order to explore the different effects of bioactive components of okra seed oil, peanut oil, and walnut oil on their antioxidant activities, the hydroxyl radical scavenging activity (*Y*_1_), ABTS radical scavenging activity (*Y*_2_), and reducing power (*Y*_3_) of oil were taken as dependent variables in this study. With the contents of total phenols (*X*_1_), total tocopherols (*X*_2_), α‐tocopherols (*X*_3_), γ‐tocopherols (*X*_4_), and δ‐tocopherols (*X*_5_) used as independent variables, a stepwise regression analysis was performed, and multiple linear regression equations (I, II, III) were established in this study (Table 7). In models (I, II, and III), F values of 104.41, 1708.70, and 223.76 were calculated, with determination coefficients (*R* ^2^) of .8818, .9993, and .9739 obtained, respectively, indicating that these models have statistical significance and ideal effects. The regression equations of relationships of hydroxyl radical scavenging activity, ABTS radical scavenging activity, and reducing power of oil and its contents of total phenols, total tocopherols, α‐tocopherols, γ‐tocopherols, and δ‐tocopherols are shown as follows: *Y*_1_ = 48.2985 + 2.0462*X*_1,_ *Y*_2_ = 0.9792*X*_1_ + 4.4447*X*_2_ + 7.1784*X*_4_, and *Y*_3_ = 0.0568 + 0.0169*X*_1_ − 0.0463*X*_4_. It can be seen that there are extremely significant correlations between the scavenging activities (hydroxyl radicals and ABTS radicals and reducing power) of vegetable oils and their contents of total phenols, total tocopherols, and γ‐tocopherols.

**TABLE 7:** Multiple regression analysis between antioxidant activities and bioactive components of okra seed oil, walnut oil, and peanut oil.

Equation	Variable	Regression coefficient	Standard regression coefficient	Regression equation	*t*‐value	*p* value	Significance
I	Intercept	48.2985	0.0000	*Y*_1_ = 48.2985 + 2.0462*X*_1_	11.44	<.0001	**
	*X*_1_	2.0462	0.8517		5.86	<.0001	**
II	*X*_1_	0.9792	0.3371	*Y*_2_ = 0.9792*X*_1_ + 4.4447*X*_2_ + 7.1784*X*_4_	7.92	<.0001	**
	*X*_2_	4.4447	0.3929		15.99	<.0001	**
	*X*_4_	7.1784	0.2835		6.82	<.0001	**
III	Intercept	0.0568	0.0000	*Y*_3_ = 0.0568 + 0.0169*X*_1_ − 0.0463*X*_4_	8.33	<.0001	**
	*X*_1_	0.0169	1.4866		9.78	<.0001	**
	*X*_4_	−0.0463	−0.5398		−3.55	.0040	**

** The variable regression is extremely significant, *p* < .01.

## 4 CONCLUSION

This study has shown that okra seed oil is rich in beneficial components (including unsaturated fatty acids, phenols, tocopherols, and mineral elements) and presents strong antioxidant activities, laying a foundation for the development of okra seed oil as an edible oil. Also, this study has proposed that there is a strong correlation between the bioactive components of okra seed oil and its antioxidant activity, thus providing a theoretical basis for solving the problem of oil oxidative rancidity. All the results obtained in this study show that okra seeds could be used as a superior oil resource, containing many natural antioxidants and presenting significant health benefits, thus showing good development and application prospects in such fields as food, maquillage, and pharmacy.

## AUTHOR CONTRIBUTIONS

Gangjun Guo: Conceptualization, Methodology, Investigation, Analysis and interpretation of the data, Writing – original draft, Writing – review & editing. Wenting Xu: Analysis and interpretation of the data, Writing – original draft, Writing – review & editing. Haiqing Zhang: Writing – review & editing. Xiaojing Hu: Writing – review & editing. Yuqin Chen: Writing – review & editing. Xiyong He: Supervision, Funding acquisition. Kechang Huang: Writing – review & editing. Shangxuan Ma: Methodology, Investigation. Jiarong Fu: Methodology, Investigation.

## CONFLICT OF INTEREST STATEMENT

The authors declare no conflicts of interest.

## Data Availability

The data that support the findings of this study are available on request from the corresponding authors.
